# Will savings from biosimilars offset increased costs related to dose escalation? A comparison of infliximab and golimumab for rheumatoid arthritis

**DOI:** 10.1186/s13075-019-2022-8

**Published:** 2019-12-12

**Authors:** Jeffrey R. Curtis, Fenglong Xie, Jonathan Kay, Joel D Kallich

**Affiliations:** 1 University of Alabama at Birmingham, 510 20th Street South, Birmingham, AL 35294 USA; 2 UMass Memorial Medical Center and University of Massachusetts Medical School, Worcester, USA; 3 Massachusetts College of Pharmacy and Health Sciences University, Boston, USA

**Keywords:** Infliximab, Golimumab, Biosimilar, Dose escalation, Rheumatoid arthritis

## Abstract

**Introduction:**

Biosimilar infliximab has the potential for appreciable cost savings compared to its reference biologic, but dose escalation is common and increases costs. We compared frequency of dose escalation and associated Medicare-approved amount so as to determine the break-even point at which infliximab dose escalation would offset the cost savings of using a biosimilar, referent to alternatively using golimumab.

**Methods:**

We studied Medicare enrollees with rheumatoid arthritis (RA) initiating infliximab or golimumab. Frequency of dose escalation was summarized descriptively over 18 months, as were Medicare-approved amounts for reimbursement. Analyses were repeated conditioning on high adherence (i.e., non-discontinuation, > 10-week gap). Multivariable-adjusted logistic regression and mixed models evaluated factors associated with infliximab dose escalation.

**Results:**

A total of 5174 infliximab and 2843 golimumab initiators were observed. Dose escalation was rare for golimumab (5%) but common for infliximab (49%), and was even more common (72%) for infliximab among patients who persisted on treatment. Regardless of dose escalation, the adjusted least square mean dollar amounts were appreciably higher for golimumab ($28,146) than for infliximab ($21,216) and greater among persistent patients (cost difference $9269, favoring infliximab). Only when patients escalated infliximab to ≥ 8 mg/kg every 6 weeks was golimumab IV at break-even or less expensive. After controlling for multiple factors, physician ownership of the infusion center was associated with greater likelihood of infliximab dose escalation (odds ratio = 1.25, 95% CI 1.09–1.44).

**Conclusion:**

Despite frequent dose escalation with infliximab that often increase its dose by threefold or more, the savings from the current price of its biosimilar substantially offsets the costs of an alternative infused TNFi biologic for which no biosimilar is available.

## Introduction

In the United States (US), the Biologics Price Competition and Innovation (BPCI) Act of 2009 created an abbreviated pathway for the Food and Drug Administration (FDA) to approve biological products that have been demonstrated to be structurally and functionally “highly similar” to an already approved biologic without clinically meaningful differences [1, 2]. As of September 2019, 23 such “biosimilars” have been approved in the US, offering the potential to provide meaningful cost savings for treating chronic diseases such as rheumatoid arthritis (RA) for which long-term use of costly biologics is common [3]. Indeed, the annual global sales for TNF inhibitors (TNFi) was $43.4 billion in 2017 [4]. Of the 23 FDA-approved biosimilars, 9 are TNF inhibitors (TNFi): 4 adalimumab biosimilars, 2 etanercept biosimilar, and 3 infliximab biosimilars. However, only 2 of the 3 infliximab biosimilars are currently marketed in the US; because of patent-related issues, the adalimumab and etanercept biosimilars are not yet available.

The context in which biosimilars compete with their reference products, and its associated impact on cost, is highly dependent on local and regional policies and payers. For example, in the European Union, as of September 2019, 55 of 61 biosimilars approved by the European Medicines Agency were commercially available [5]. In some European Economic Area countries such as Norway, hospital-administered medications are purchased by a single payer using a competitive tender system. By this process, the single payer can negotiate aggressively for discounted acquisition costs, which has realized savings of up to 70% for biosimilar infliximab compared to its reference product [6]. Alternatively, competition created by the introduction of a biosimilar may result in savings by driving down the price of the bio-originator. In late 2018, AbbVie won the Swedish national tender for adalimumab by dropping its price for the bio-originator by 80% [7]. Thus, in countries in which there are single payer systems and “winner-take-all” competitive bidding for drug acquisition, the promise of significant cost savings with the availability of biosimilars is in fact being realized [8]. However, in the US healthcare system, in which payers and their pharmacy benefit managers may negotiate undisclosed reductions in drug prices based upon discounts and rebates from manufacturers, the availability of biosimilar infliximab has resulted in only a 22% reduction of the average selling price (ASP) of biosimilar infliximab compared to that of its reference product [9, 10]. Medicare is the largest single payer of biologic medications in the US, yet the Medicare program has a much more limited ability to negotiate price compared to its European counterparts, making the magnitude of biosimilar discounting important from a policy and health economic perspective.

A unique aspect of some biologics used to treat RA is the potential for on-label dose escalation. This might take the form of an increase either in the dose administered or in the dosing frequency, or both. Infliximab, a TNFi indicated for the treatment of a number of inflammatory diseases, can be escalated in this way. Given past findings of relatively frequent infliximab dose escalation, we conducted an analysis of Medicare data to compare the frequency of dose escalation and the total Medicare-approved dollar amount for infliximab compared to intravenous golimumab, the only other TNFi medication administered by infusion and which has relatively similar disease indications. The purpose of the analysis was to test the hypothesis that dose escalation of infliximab might offset the potential savings from using biosimilar infliximab, contrasted with the use of an alternative TNFi (golimumab) for which a biosimilar is neither available nor expected in the near future.

## Methods

### Overview

We constructed a cohort of new infliximab and golimumab IV users to evaluate descriptively the frequency of dose escalation for each drug, persistence on treatment, and associated Medicare-approved dollar amounts for reimbursement. We further examined factors associated with infliximab dose escalation to evaluate whether provider-level variability was a factor that influenced dose escalation, given that physician prescribing practices are potentially modifiable. Finally, we used the results from these analyses to populate a hypothetical cost model that evaluated the extent of infliximab dose escalation which would be required to offset the dollar savings derived from use of biosimilar infliximab, referent to an alternative strategy of instead using golimumab IV, an infused TNFi for which no biosimilar is available. Our analysis is thematically similar to an ongoing, phase 4 comparative pragmatic trial that is examining the frequency of dose escalation, persistence on treatment, and other outcomes associated with use of golimumab IV versus infliximab in RA (clinicaltrials.gov NCT02728934; https://clinicaltrials.gov/ct2/show/NCT02728934).

### Cohort eligibility

Using the Centers for Medicare and Medicaid Services (CMS) fee-for-service Medicare data from 2012 to 2016, we assembled two cohorts of RA patients initiating either infliximab or intravenous golimumab to compare dose escalation, persistence on treatment, and amount paid by the Medicare program. To be eligible for analysis, patients must have had a claim for reimbursement for RA, identified using ICD9-CM diagnostic codes (ICD9: 714.0, 714.2, 714.81; ICD10: M05.*, M06.*) from rheumatologists. The date of first administration was defined as the index date for each patient, and the 12-month period preceding the index date was defined as “baseline.” All patients must have had their index date in 2013 or later, given that was the year of golimumab IV licensure in the US for RA. Patients were required to be new users of each of these therapies, with no claims for reimbursement of these drugs, in both the baseline period and all preceding available data (if more was available). Additional covariates of interest (e.g., use of methotrexate (MTX) and glucocorticoids (GC)) were examined during the 12-month baseline period. All patients were required to have Medicare part A, B, and D coverage during the 12-month baseline period and for the 78 weeks after the index date. Patients were permitted to contribute (at most) one exposure episode to the infliximab cohort and/or to the golimumab cohort, if they met all criteria above.

### Biologic exposure and definitions of dose escalation

Infliximab and golimumab for intravenous use (IV) were identified using Healthcare Common Procedure Coding System (HCPCS) codes (J1745 for bio-originator infliximab, Q5102 for biosimilar infliximab-dyyb, and J1602 for golimumab IV; codes Q5103 and Q5104 were not used until April 1, 2018, and thus were not included in these data), as well as using National Drug Codes (NDCs) [11, 12]. NDCs for infliximab or golimumab IV drug dispensation followed by the HCPCS code for its administration within 7 days were consolidated into a single claim. To treat RA, infliximab is typically administered at an initial dose of 3 mg/kg and golimumab IV at a fixed dose of 2 mg/kg. Because infliximab is dispensed in 100 mg vials and golimumab IV in 50 mg vials, each dose administered was rounded up to the nearest 100 mg increment (for infliximab) or 50 mg increment (for golimumab IV). RA patients who initiated therapy either at an implausibly low starting dose (e.g., ≤ 100 mg infliximab or ≤ 50 mg golimumab IV) or at an implausibly high starting dose (≥ 1000 mg infliximab or ≥ 350 mg golimumab IV) were excluded. Fewer than 1% of all initiations were excluded for this reason.

A dose increase was defined as an increase of ≥ 100 mg (infliximab) or ≥ 50 mg (golimumab IV) compared to the initial dose. The maximum plausible dose of infliximab and golimumab IV allowable (after the starting dose) was capped at 1500 mg and 600 mg, respectively. Increase in frequency was defined by any pairwise interval shorter than the usual 8-week dosing schedule common to both drugs, after the initial loading dose (0, 2, and 6 weeks for infliximab, and 0 and 4 weeks for golimumab IV). The pairwise intervals between infusions were rounded up to the nearest week to be conservative. For example, an interval of 50–62 days between infusions would be considered compatible with an 8-week dosing interval. Dose escalation was defined as either a dose increase or an increase in dosing frequency. To define dose escalation conservatively, and because irregular patient scheduling or other factors might lead to what would incorrectly appear to be a dose increase, we required that dose increase or dosing interval shortening occurs at two consecutive infusions in order to satisfy the dose escalation criteria. A subgroup analysis was performed on patients who remained on therapy (see definition below) to evaluate both the frequency of dosing and the Medicare-approved dollar amounts associated with each of the two infused TNFi among these patients. A sensitivity analysis was also conducted that required only a single dose increase or frequency shortening, rather than require an increase at two consecutive infusions, as in the main analysis.

### Outcomes of interest

The primary outcomes of interest were the frequency of dose escalation and the amount paid by Medicare through 78 weeks (day 546) following treatment initiation. Non-persistence (i.e., treatment discontinuation) was a secondary outcome, defined by either a ≥ 10-week gap without the medication, or switching to another biologic treatment for RA. A composite outcome of time to either discontinuation or dose escalation was also examined. Medication “payments” were obtained directly from the Medicare raw data as the Medicare-approved dollar amount which is the full payment for covered services for providers who accepts assignment, as listed on each infusion claim. The Medicare-approved dollar amount is the maximum amount approved by Medicare for which a medical service, including infused medications, can be reimbursed. Medicare typically pays 80% of this amount; the rest is collected from either the patient as coinsurance or a supplemental insurance policy such as Medigap.

### Statistical analysis

Descriptive statistics were used to compare the infliximab and golimumab IV cohorts at baseline. Patient covariates of interest included age, sex, race, disability as the reason for Medicare eligibility (given that RA itself is a common reason for qualifying for Medicare), dual eligible beneficiaries (i.e., eligible for both Medicare and Medicaid, a program for individuals and families with low income and limited resources), comorbidities such as chronic pulmonary disease, RA-related treatments (e.g., methotrexate, NSAIDs) and other chronic medications, and measures of healthcare utilization including number of physician visits and inpatient hospitalization.

Rheumatology providers were assigned uniquely to each patient based on that office visit with an RA diagnosis code, which was most proximate to and prior to the date of infliximab or golimumab IV initiation. Physician ownership of the infusion center was assigned based on whether the physician billed under his or her own NPI or was part of a group that billed for infusions. A Data Use Agreement governed use of all of the CMS data, and the analysis was approved by the University of Alabama at Birmingham Institutional Review Board. All analyses were performed in SAS 9.4.

#### Cohort analysis evaluating persistence, Medicare-approved amounts, and factors associated with infliximab dose escalation

Kaplan-Meier curves were used to evaluate drug discontinuation through week 78, and separately, a composite outcome of discontinuation or dose escalation. Logistic regression was used to generate a propensity score for receipt of infliximab versus golimumab and to control for differences in baseline factors. The propensity score was then used to create inverse probability of treatment weights (IPTW) and applied to a generalized linear model procedure with a gamma distribution to calculate adjusted least square means of infliximab and golimumab IV users.

Among both bio-originator and biosimilar infliximab users only, a separate logistic regression model was created to evaluate the likelihood that patients were dose escalated on infliximab (referent to not escalated) and to evaluate baseline factors that might be associated with dose escalation. Mixed models were used to account for the clustered nature of the data (i.e., patients are treated within rheumatology practices). A variety of covariates were examined that were hypothesized to be associated with the the likelihood that a physician might dose escalate, including physician characteristics (e.g., ownership of infusion center), and patient factors including age, sex, race, disability, dual eligibility (commonly a proxy for lower income), comorbidities, conventional synthetic DMARDs, oral glucocorticoids, NSAIDs, opioids, anti-depressants, and proxies for health-seeking behaviors and screening including use of statins, anti-hypertensive medications, lipid lowering medications, and breast cancer screening. Given the low frequency of dose escalation for golimumab IV, no modeling was performed for golimumab IV dose escalation.

#### Break-even analysis of infliximab dose escalation versus use of golimumab IV

A hypothetical modeling scenario was run that compared dose-escalated infliximab versus golimumab IV, using the actual Medicare-approved amounts by Medicare in the first quarter of 2016, to evaluate the extent of infliximab dose escalation that would be required to offset the higher Medicare-approved amount for golimumab. This modeling took into account a range of potential discounts for biosimilar infliximab and assuming one of several body weights (60, 70, 80, and 120 kg).

#### Uptake of biosimilar infliximab through December 2017

Finally, to examine more contemporary data to report descriptively on the uptake of biosimilar infliximab, we obtained data from the CMS Part B National Summary Data File [13]. These data describe the use of various healthcare services and associated Medicare-approved amounts for medical procedures (including infusion therapies) by CMS’ Medicare program, through December 31, 2017.

## Results

Through the end of 2016, among 386,997 administrations of infliximab, fewer than 1% were for the biosimilar infliximab-dyyb (Q5102), and the median price per 100 mg infliximab vial was $829. After applying the inclusion and exclusion criteria (Additional file 2: Figure S1), 5174 patients initiated infliximab, and 2843 patients initiated golimumab IV. The characteristics both of patients who received treatment and of the clinicians who prescribed the medications were relatively similar for infliximab and golimumab (Table 1). However, prescribers of golimumab IV were older and had been in practice longer than prescribers of infliximab, and were more likely to own their own infusion practice. Golimumab-treated patients were slightly older (70.0 versus 68.6 years), less likely to be dual eligible, and more likely to receive monotherapy (i.e., without methotrexate or other conventional synthetic DMARDs).

**Table 1:** Baseline characteristics of patients initiating golimumab or infliximab and characteristics of their prescribing clinicians

	Golimumab (*N* = 2843)	Infliximab (*n* = 5174)	SMD
Physician-specific factors			
Female sex	679 (23.9%)	1368 (26.4%)	0.06
Age in years, mean (STD)	*52.74 (9.43)*	*51.40 (9.29)*	*0.14*
Years in practice	*20.75 (10.86)*	*18.68 (10.81)*	*0.19*
Ownership of infusion center	*2528 (88.9%)*	*4162 (80.4%)*	*0.24*
Office-based practice**	2230 (78.4%)	3906 (75.5%)	0.07
Type of employment			
Federal government	108 (3.8%)	185 (3.6%)	0.01
Group practice	1298 (45.7%)	2237 (43.2%)	0.05
Local government	180 (6.3%)	409 (7.9%)	0.06
Medical school	79 (2.8%)	110 (2.1%)	0.04
Other	567 (19.9%)	1187 (22.9%)	0.07
Solo practice	611 (21.5%)	1046 (20.2%)	0.03
Patient-specific factors			
Demographics			
Age in years, mean (STD)	*70.01 (8.90)*	*68.66 (9.47)*	*0.15*
Female	2287 (80.4%)	3988 (77.1%)	0.08
White	2349 (82.6%)	4233 (81.8%)	0.02
Dual eligible for Medicare and Medicaid	*348 (12.2%)*	*886 (17.1%)*	*0.14*
Disability according to Medicare as original reason for Medicare eligibility	1004 (35.3%)	1676 (32.4%)	0.06
Comorbidity diagnoses, %			
Myocardial infarction	137 (4.8%)	226 (4.4%)	0.02
Coronary heart disease	554 (19.5%)	977 (18.9%)	0.02
Peripheral vascular disease	217 (7.6%)	389 (7.5%)	0.00
Chronic pulmonary disease	771 (27.1%)	1359 (26.3%)	0.02
Peptic ulcer disease	52 (1.8%)	86 (1.7%)	0.01
Diabetes	658 (23.1%)	1168 (22.6%)	0.01
Renal disease	287 (10.1%)	462 (8.9%)	0.04
Malignancy	195 (6.9%)	331 (6.4%)	0.02
Fibromyalgia	591 (20.8%)	978 (18.9%)	0.05
RA and other medications, %			
Methotrexate	*1642 (57.8%)*	*3595 (69.5%)*	*0.25*
Other conventional DMARDS	*1085 (38.2%)*	*2386 (46.1%)*	*0.16*
Oral glucocorticoids	2039 (71.7%)	3914 (75.6%)	0.09
NSAIDs	1299 (45.7%)	2453 (47.4%)	0.03
Opioid	2030 (71.4%)	3585 (69.3%)	0.05
Statin	1315 (46.3%)	2219 (42.9%)	0.07
Other lipid lowering drug	257 (9.0%)	418 (8.1%)	0.03
Anti-hypertensive drug	2202 (77.5%)	3822 (73.9%)	0.08
Anti-depressant drug use	1283 (45.1%)	2181 (42.2%)	0.06
Healthcare utilization			
Number of physician visits, Mean (STD)	17.89 (9.35)	16.96 (9.17)	0.10
Any hospitalization	604 (21.2%)	1027 (19.8%)	0.03
Colon cancer screening	442 (15.5%)	931 (18.0%)	0.07
Breast cancer screening	1197 (42.1%)	2097 (40.5%)	0.03

*SMD* standardized mean difference. A SMD > 0.10 (italicized) is indicative of a potentially important difference

Data shown as mean (standard deviation) or n (%)

*Two consecutive infusions with a dose increase, or frequency increase, were required to satisfy this definition

**Rather than hospital-based practice, research, or other/missing designations

**Fig. 1:**
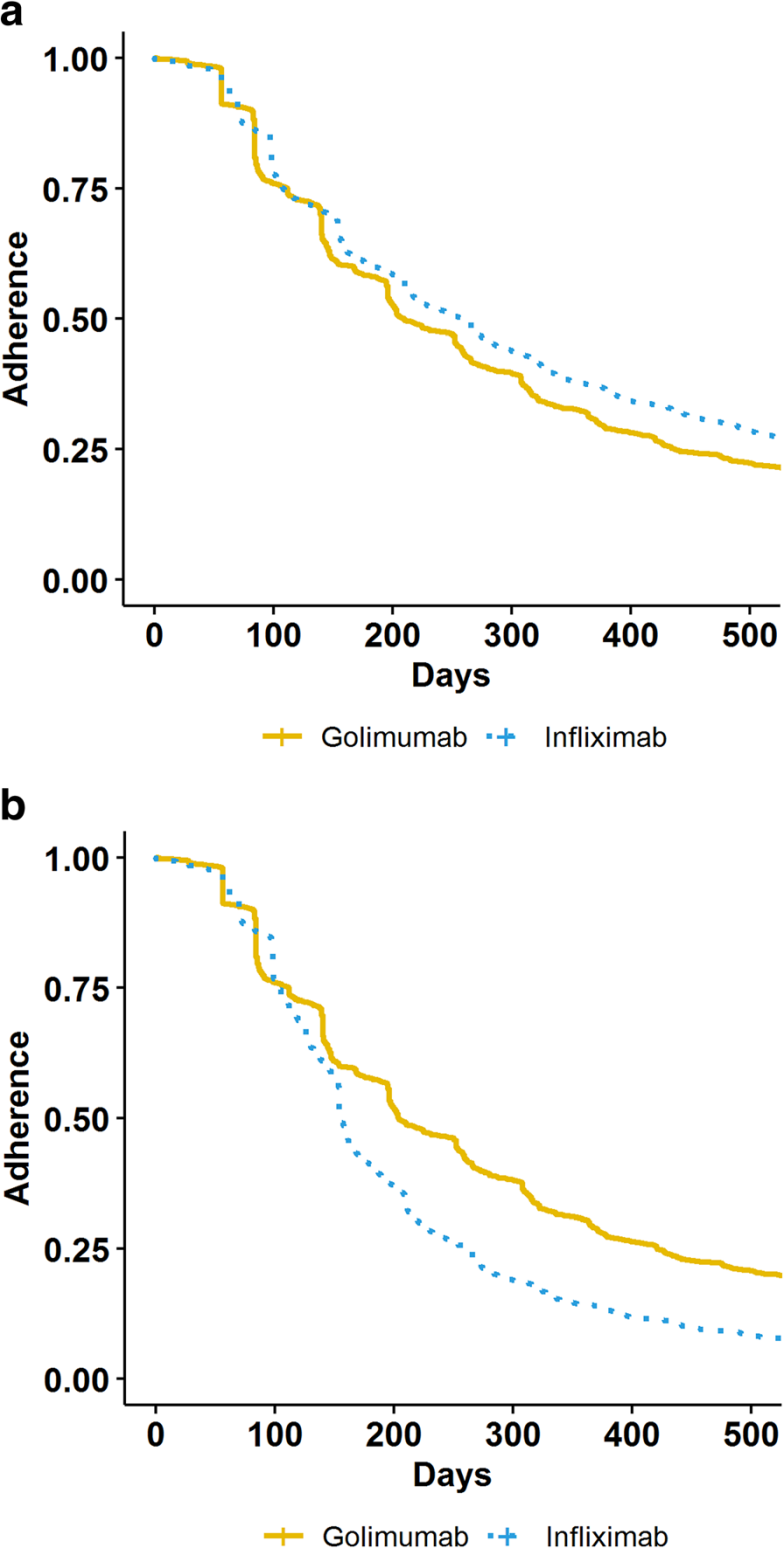
**a** KM curves for persistence with infliximab and intravenous golimumab. Note: non-persistence defined as a gap > 10 weeks in therapy. **b** KM curve for the composite outcome of persistence or dose escalation of infliximab and intravenous golimumab. Note: non-persistence defined as a gap > 10 weeks in therapy

Overall non-persistence with golimumab IV was worse than for infliximab (Fig. 1a, *p* < 0.0001). However, when a composite outcome of non-persistence or dose escalation was considered, the time to the composite outcome was shorter for the infliximab users (Fig. 1b, *p* < 0.0001). Approximately three quarters of infliximab users discontinued or dose escalated by 18 months.

Fewer than 5% of golimumab IV patients dose escalated, whereas approximately half of infliximab patients dose escalated, through 18 months. Comparing patients who dose escalated infliximab or golimumab IV to those who did not (Additional file 1: Table S1) revealed few characteristics that differentiated infliximab users who dose escalated from those who did not. There were several differences between golimumab IV users who underwent dose escalation and those who did not and their treating clinicians. The clinicians who dose escalated patients were somewhat older, had been in practice longer, and were more likely to be in office-based practice. Patients who had a higher prevalence of certain comorbidities (e.g., chronic pulmonary disease, peripheral vascular disease) were more likely to undergo dose escalation.

As shown in Table 2, the mean [SD] costs paid by Medicare over the first 18 months of treatment were significantly greater for golimumab IV ($28,146 [16,030]) than for infliximab ($21,216 [15,819]), a 33% difference. When the analysis was restricted to the minority of patients who persisted on therapy through 18 months with no gap in treatment > 10 weeks, least square mean costs were 27% higher for golimumab IV ($43,940) than for infliximab ($34,671).

**Table 2:** Dose escalation and IPTW-adjusted Medicare-approved amount for the biologic medication through week 78, in both the as-observed and persistent cohorts

	Infliximab (*N* = 5174)	Golimumab (*N* = 2843)	*p* value
Overall cohort			
Dose escalation*, %	49.46	4.89	< 0.0001
Dose increase, %	39.49	3.17	< 0.0001
Frequency increase, %	29.15	1.79	< 0.0001
Discontinuation, %	73.33	79.85	< 0.0001
Biologic Medicare-approved amounts, day 0–546, $			
All biologics**			
LS mean (95% CI)	26,934 (26,441–27,435)	35,512 (34,849–36,187)	< 0.0001
Index biologic			
LS mean (95% CI)	21,216 (20,737–21,706)	28,146 (27,497–28,810)	< 0.0001
Biologic Medicare-approved amounts, day 183–546, $			
All biologics**			
LS mean (95% CI)	16,401 (15,699–17,135)	20,512 (19,615–21,450)	< 0.0001
Index biologic			
LS mean (95% CI)	11,488 (10,813–12,205)	14,055 (13,213–14,951)	< 0.0001
Persistent cohort (no switch or gap > 10 weeks)	*N* = 1380	*N* = 573	
Dose escalation*, %	71.96	7.85	< 0.0001
Dose increase, %	58.55	5.24	< 0.0001
Frequency increase, %	45.00	2.97	< 0.0001
Biologic Medicare-approved amounts, day 0–546, $			
Index biologic			
LS mean (95% CI)	34,671 (33,891–35,470)	43,940 (42,849–45,058)	< 0.0001
Biologic Medicare-approved amounts, day 183–546, $			
Index biologic			
LS mean (95% CI)	22,877 (22,301–23,467)	27,454 (26,692–28,238)	< 0.0001

*LS* inverse probability treatment (IPTW)-weighted least square mean

IPTW weighting controlled for patient age, sex, race, number of physician visits, number of prior biologic DMARDS, methotrexate use, statin use, reason for eligible for Medicare, and 55 of the CCS categories (Additional file 1: Table S2) which were significant in univariate analyses in their association with cost from day 183–546

*Dose and frequency increases are not mutually exclusive. Note that two consecutive infusions were required to meet definition for dose and frequency escalation

**Includes cost of both the index therapy (infliximab or golimumab) and any subsequent biologic switch through day 546. Costs from day 183–546 were shown to be able to describe costs following the loading period for each drug

**Fig. 2:**
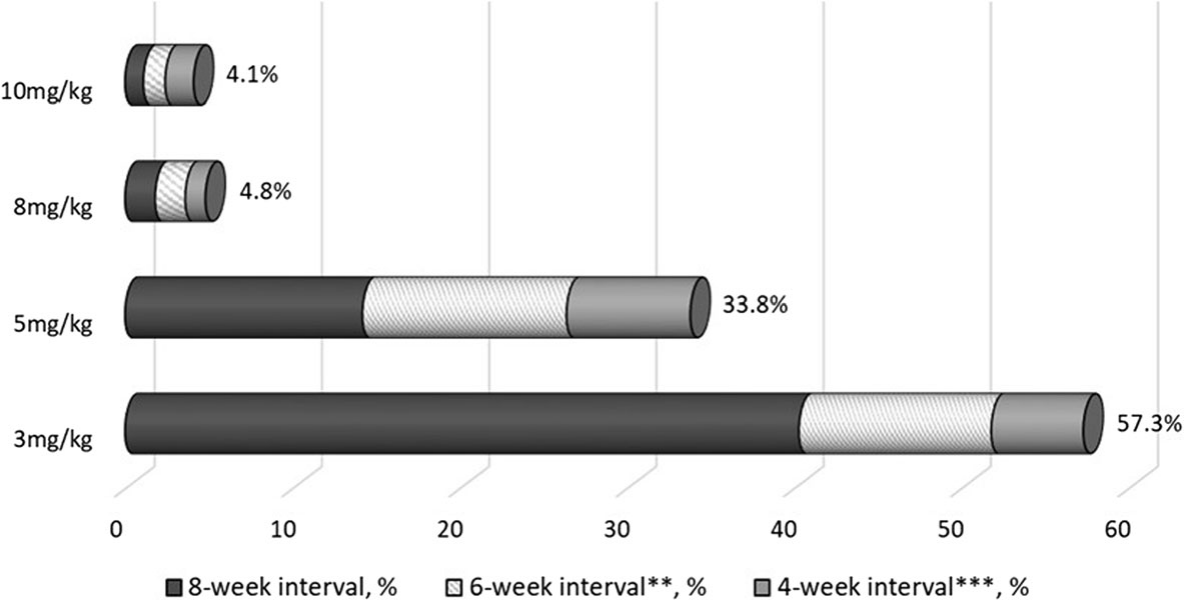
Maximum dose and frequency of infliximab administered through 18 months* (*n* = 4502). *Restricted to patients with a consistent dose for all 3 infusions, throughout the induction period (week 0, 2, 6), at starting doses of 200, 300, or 400 mg, representing 87% of all 5174 patient in the infliximab cohort. All patients were assumed to be starting at a dose of 3 mg/kg, every 8 weeks. In this analysis, only a single dose and frequency increase was required, unlike in the main analysis where two consecutive infusions were required to meet the dose escalation definition. **Infusion interval for q6w infusion ranges from 36 to 48 days, inclusive (42 ± 6 days). ***Infusion interval for q4w infusion ranges from 22 to 34 days, inclusive (28 ± 6 days)

The sensitivity analysis, which required only a single dose increase or dosing frequency shortening after the baseline dose and classified all patients in mutually exclusive categories based on their maximal dose and dosing frequency for any infusion through 18 months, is shown in Fig. 2. Only about 40% of infliximab-treated patients were observed to continue on 3 mg/kg at an every 8-week dosing interval. One third (33.9%) of patients increased their dose to 5 mg/kg, and 8-9% increased their dose to ≥ 8 mg/kg or 10 mg/kg.

After multivariable adjustment, physician ownership of the infusion center was associated with a 25% greater likelihood of infliximab dose escalation (Table 3). Older patient age, female sex, presence of chronic pulmonary disease, being disabled, and being a dual eligible beneficiary were associated with a lower likelihood of dose escalation.

**Table 3:** Baseline factors associated with infliximab dose escalation (*n* = 5174 initiators)

Factor	Adjusted* odds ratio (95% CI)
Physician ownership of infusion practice	1.25 (1.09–1.44)
Patient age (5 year increments)	0.93 (0.89–0.96)
Male sex	1.20 (1.04–1.40)
Chronic pulmonary disease	0.84 (0.74–0.95)
Disability	0.84 (0.72–0.98)
Dual eligibility	0.79 (0.66–0.94)

*Also adjusted for race, use of other conventional synthetic DMARDs, use of oral glucocorticoid, NSAIDs, opioids, statins, anti-hypertensive medications, lipid lowering medications, anti-depressants, and breast cancer screening, none of which were significant

**Fig. 3:**
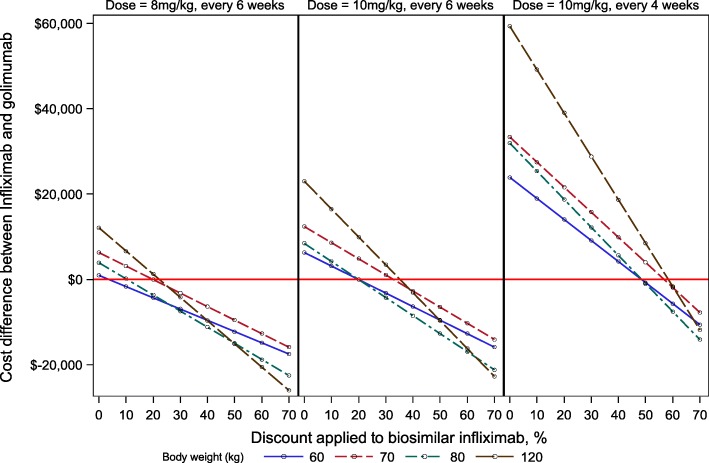
Break-even dose and frequency of infliximab versus stable-dose intravenous golimumab, across a range of discounting assumed for biosimilar infliximab. Note: positive numbers reflect greater costs for infliximab, and negative numbers reflect a cost savings for use of infliximab, compared to golimumab IV

In the modeling scenario used to evaluate the dose and frequency of infliximab that would be required to offset the higher costs of golimumab IV, all dosing frequencies of infliximab at doses of either 3 mg/kg or 5 mg/kg (with no discounting) yielded lower annual costs when compared to golimumab IV. For infliximab doses ≥ 8 mg/kg, results are shown in Fig. 3 (with the underlying data for all key dose and frequency combinations available in Additional file 1: Table S1). Positive numbers (above the zero line on the *y* axis) reflect higher costs for infliximab, and negative numbers reflect lower costs for infliximab, referent to golimumab IV, and are shown to four body weights. Infliximab at 8 mg/kg became less costly than golimumab IV once discounts of greater than 25% were available. At 10 mg/kg dosed every 6 weeks, however, the cost of infliximab was lower than that of golimumab IV only if discounts greater than of approximately 30% or more were applied (depending on body weight); at 10 mg/kg dosed every 4 weeks, discounts of 50% or more would be needed in order to yield a lower cost for infliximab compared to that of golimumab IV. Finally, based on aggregate part B data available from CMS for January 2017 through December 2017, the proportion of Medicare-approved charges for biosimilar infliximab as a fraction of all infliximab Medicare allowed charges had grown to 5.1% (Q5101, 2.6%; Q5102, 2.5%).

## Discussion

In this analysis of real-world data from the US Medicare program, we found that infliximab dose escalation was common, occurring in approximately one half of patients during the first 18 months of treatment. However, given the appreciately higher drug-related cost of golimumab IV relative to infliximab, treatment with infliximab became more expensive only if patients escalated the infliximab dose to at least 8 mg/kg. Even in that circumstance, a 20% or greater discount to the amount reimbursed for infliximab largely offset the increased expense of infliximab incurred by escalating the dose up to 8 mg/kg every 6 weeks. Given the 21% lower average sales price (ASP) of biosimilar infliximab-dyyb, as compared to the ASP of bio-originator infliximab, in Q3 2019 (Additional file 3: Figure S2) [10], even infliximab dose at 8 mg/kg q 6 weeks should be approximately neutral or cost saving compared to golimumab IV. However, at 10 mg/kg dosed every 4 weeks, infliximab would have to be discounted by 50–60% to achieve parity with the cost of golimumab IV, a circumstance that is perhaps unlikely to occur in the US in the near future.

We found that dose escalation of infliximab is common in this cohort of RA patients. Indeed, only about 40% of patients remained on their starting dose, which is typically 3 mg/kg q 8 weeks for RA. While the operational definitions of dose escalation may vary from study to study and may differ somewhat according to the data source, approximately 30–60% of RA patients in the US typically dose escalate [14–17]. While one might contend that infliximab dose escalation can achieve incremental clinical benefit, most published studies that have examined clinical outcomes have not found improved control of disease activity with infliximab compared to other TNFi therapies, even with its potential for dose and frequency escalation [18–21].

Perhaps of importance, we found that patients treated by physicians who owned the infusion practice were more likely to undergo dose escalation, similar to prior observations [22]. We also observed that dose escalation was associated with several patient characteristics. Younger individuals, men, and those who did not have chronic pulmonary disease were more likely to dose escalate. Those who were dual eligible and disabled were less likely to dose escalate, which may reflect the fact that these patients who also have Medicaid coverage (i.e., because they are dual eligible) have a broader range of treatment choices available to them, including self-injected and oral targeted therapies (e.g., tofacitinib). Previously published studies have shown that despite infliximab dose escalation, patients typically achieve clinical outcomes comparable to those with other biologics used to treat RA [23, 24], yet dose escalation appears to confer a greater risk of serious infections [25]. Dose escalation also results in increased costs, both financial direct costs, as described in this and other studies [16, 18, 26], and indirect costs including the time burden to patients of more frequent and longer duration of infusions. Thus, despite the availability and potential cost savings of biosimilars, infliximab dose escalation beyond 5 mg/kg is probably not a prudent course of treatment for most RA patients compared to switching to alternative treatment options.

The strengths of our study include a relatively large sample size of patients receiving care in the US. The generalizability of the US Medicare population to older individuals is excellent, in that Medicare covers approximately 94% of the US population age ≥ 65 years. Moreover, we were able to examine physician ownership of the infusion center that might influence motivation to continue to keep people on an intravenous therapy and to escalate the dose of the infused medication. Nevertheless, our results must be interpreted in light of our study design. We lacked information on the clinical reasons for treatment discontinuation (e.g., lack of clinical response, safety/tolerability) and dose escalation. We did not study safety- or tolerability-related factors, although infliximab has been commonly associated with mild infusion reactions and infrequently with severe infusion reactions [27].

Our data extended through the end of 2016. Biosimilar infliximab-dyyb was first marketed in the US on November 28, 2016 [28], and we observed minimal use of biosimilar infliximab through the end of that year. However, it likely has increased appreciably in a more contemporary time in 2018 [29]. Moreover, as of April 1, 2018, the Medicare program changed the coding and reimbursement for biosimilar infliximab, replacing the HCPCS code Q5102 under which all biosimilar infliximab products were grouped with individual HCPCS codes Q5103 for biosimilar infliximab-dyyb and Q5104 for biosimilar infliximab-abda, which allowed each product to have its own ASP [30]. Since a biosimilar is reimbursed at its own ASP plus 6% of the ASP of its reference product, the introduction of unique HCPCS codes for individual biosimilars creates price competition among biosimilars and has resulted in further reductions in the cost of infliximab, both bio-originator and biosimilars (Additional file 3: Figure S2) [30]. However, unlike the discounts of up to 70% that have been achieved for biosimilar infliximab in the Norwegian tender system [6], price reductions sufficient to offset the higher costs of infliximab dose escalation to 10 mg/kg infused every 4 or 6 weeks may not be attainable in the US.

## Conclusion

The costs associated with dose escalating infliximab to 10 mg/kg every 4 or 6 weeks are substantial and likely offset even appreciable dose savings associated with biosimilars. Under those circumstances, use of an alternative medication (e.g., golimumab IV) is likely to be less expensive and, on average, has been shown to result in similar clinical outcomes. For all other lower infliximab doses and frequencies, however, the costs associated with dose escalation likely would be offset by the savings associated with use of biosimilar infliximab. Finally, although cost is an important consideration in selecting among biologics, other clinical factors (e.g., shorter infusion time, lower incidence of hypersensitivity infusion reactions, and incidence of serious infections) should be considered when selecting a specific biologic agent.

## Supplementary information

**Additional file 1:** **Table S1.** Baseline Patient and Physician Characteristics, stratified for infliximab vs. golimumab and by whether patients dose escalated*. SMD = standardized mean difference. A SMD > 0.10 (bolded) is indicative of a potentially important difference. Data shown as mean (standard deviation) or n (%). *Two consecutive infusions with a dose increase, or frequency increase, were required to satisfy this definition. **rather than hospital-based practice, research, or other/missing designations. **Table S2.** AHRQ CCS categories included in the inverse probability treatment weighting model to balance covariates between golimumab and infliximab initiators.

**Additional file 2: Figure S1.** Cohort Selection.

**Additional file 3: Figure S2.** Decrease in Average Selling Price* of Infliximab Biosimilars Over Time. *Centers for Medicare and Medicaid CMS Medicare Part B Drug Average Sales Price Report (updated September 10, 2019 from https://www.cms.gov/Medicare/Medicare-Fee-for-Service-Part-B-drugs/McrPartBDrugAvgSalesPrice/2018ASPFiles.html).

## Data Availability

The data that support the findings of this study are available from Centers for Medicare and Medicaid Services (CMS). However, the data is non-public, and access to data files is restricted to users of the DUA under authorization of CMS.

## Abbreviations

ASP: Average sales price
BPCI: Biologics Price Competition and Innovation
CMS: Center for Medicare and Medicaid Services
FDA: Food and Drug Administration
HCPCS: Healthcare Current Procedure Coding System
IPTW: Inverse probability treatment weighted
IV: Intravenous
RA: Rheumatoid arthritis
TNFi: Tumor necrosis factor inhibitor
US: United States

## References

[1] 111th Congress of the United States of America. H. R. 3590—686 - Title VII—Improving Access to Innovative Medical Therapies, Subtitle A—Biologics Price Competition and Innovation. 2010. https://www.fda.gov/downloads/drugs/ucm216146.pdf Accessed 11 Mar 2019.

[2] U.S. Food and Drug Administration. Scientific Considerations in Demonstrating Biosimilarity to a Reference Product: Guidance for Industry. 2015. https://www.fda.gov/downloads/DrugsGuidanceComplianceRegulatoryInformation/Guidances/UCM291128.pdf Accessed 10 Sept 2019.

[3] U.S. Food & Drug Administration. Biosimilar Product Information. 2019. https://www.fda.gov/drugs/developmentapprovalprocess/howdrugsaredevelopedandapproved/approvalapplications/therapeuticbiologicapplications/biosimilars/ucm580432.htm#top Accessed 11 Mar 2019.

[4] Credence Research. Tumor Necrosis Factor (TNF) Inhibitor Market Projected to Grow With Double Digit CAGR during the Forecast Period. 2018. https://www.credenceresearch.com/report/tumor-necrosis-factor-tnf-inhibitors-market Accessed 11 Mar 2019.

[5] GaBI Online. Biosimilars approved in Europe. 2011. http://www.gabionline.net/Biosimilars/General/Biosimilars-approved-in-Europe Accessed 10 Sept 2019.

[6] Mack A (2015). Norway, biosimilars in different funding systems. What works?. Generics Biosimilars Initiative J.

[7] Brennan Z. AbbVie Sees 80% Discounts in Nordic Market With New Humira Biosimilars. 2018. Regulatory Affairs Professionals Society (RAPS). https://www.raps.org/news-and-articles/news-articles/2018/11/abbvie-sees-80-discounts-in-nordic-market-with-ne Accessed 11 Mar 2019.

[8] GaBI Online. Huge discount on biosimilar infliximab in Norway 2015. http://www.gabionline.net/Biosimilars/General/Huge-discount-on-biosimilar-infliximab-in-Norway Accessed 11Mar 2019.

[9] U.S. Centers for Medicare & Medicaid Services (CMS.gov). 2018 ASP Drug Pricing Files. 2018. https://www.cms.gov/Medicare/Medicare-Fee-for-Service-Part-B-Drugs/McrPartBDrugAvgSalesPrice/2018ASPFiles.html Accessed 11 Mar 2019.

[10] U.S. Centers for Medicare & Medicaid Services (CMS.gov). 2019 ASP Drug Pricing Files. 2019. https://www.cms.gov/Medicare/Medicare-Fee-for-Service-Part-B-Drugs/McrPartBDrugAvgSalesPrice/2019ASPFiles.html Accessed 11 Mar 2019.

[11] U.S. Centers for Medicare & Medicaid Services (CMS.gov). HCPCS - General Information: Notification of Change in Medicare Coverage and Pricing Indicators for HCPCS Codes Q9994 and B4105 for In-Line Cartridge Containing Digestive Enzymes(s) for Enteral Feeding, Each. 2019. https://www.cms.gov/Medicare/Coding/MedHCPCSGenInfo/index.html?redirect=/MedHCPCSGenInfo/ Accessed 11 Mar 2019.

[12] U.S. Food & Drug Administration. National Drug Code Directory. 2019. https://www.fda.gov/drugs/informationondrugs/ucm142438.htm Accessed 11 Mar 2019.

[13] U.S. Centers for Medicare & Medicaid Services (CMS.gov). Part B National Summary Data File (Previously known as BESS) 2019. https://www.cms.gov/Research-Statistics-Data-and-Systems/Downloadable-Public-Use-Files/Part-B-National-Summary-Data-File/Overview.html Accessed 11 Mar 2019.

[14] Rahman MU, Strusberg I, Geusens P, Berman A, Yocum D, Baker D (2007). Double-blinded infliximab dose escalation in patients with rheumatoid arthritis. Ann Rheum Dis.

[15] Tang B, Rahman M, Waters HC, Callegari P (2008). Treatment persistence with adalimumab, etanercept, or infliximab in combination with methotrexate and the effects on health care costs in patients with rheumatoid arthritis. Clin Ther.

[16] Nadkarni A, McMorrow D, Patel C, Fowler R, Smith D (2017). Incidence of dose escalation and impact on biologic costs among patients with rheumatoid arthritis treated with three intravenous agents. J Comp Eff Res.

[17] Thorne C, Boire G, Chow A, Garces K, Liu F, Poulin-Costello M (2017). Dose escalation and co-therapy intensification between etanercept, adalimumab, and infliximab: the CADURA study. Open Rheumatol J.

[18] Institute for Clinical and Economic Review (ICER)**.** Evidence Report: Targeted Immune Modulators for Rheumatoid Arthritis: Effectiveness & Value. New England Comparative Effectiveness Public Advisory Council (CEPAC); 2017:64. https://icer-review.org/wp-content/uploads/2016/08/NE_CEPAC_RA_Evidence_Report_FINAL_040717.pdf Accessed 11 Mar 2019.

[19] Flouri I, Markatseli TE, Voulgari PV, Boki KA, Papadopoulos I, Settas L (2014). Comparative effectiveness and survival of infliximab, adalimumab, and etanercept for rheumatoid arthritis patients in the Hellenic Registry of Biologics: low rates of remission and 5-year drug survival. Semin Arthritis Rheum.

[20] Greenberg JD, Reed G, Decktor D, Harrold L, Furst D, Gibofsky A, et al. A comparative effectiveness study of adalimumab, etanercept and infliximab in biologically naive and switched rheumatoid arthritis patients: results from the US CORRONA registry. Ann Rheum Dis. 2012;71(7):1134–42. 10.1136/annrheumdis-2011-150573 22294625

[21] Hetland ML, Christensen IJ, Tarp U, Dreyer L, Hansen A, Hansen IT (2010). Direct comparison of treatment responses, remission rates, and drug adherence in patients with rheumatoid arthritis treated with adalimumab, etanercept, or infliximab: results from eight years of surveillance of clinical practice in the nationwide Danish DANBIO registry. Arthritis Rheum.

[22] Zhang J, Xie F, Delzell E, Chen L, Kilgore ML, Yun H (2013). Trends in the use of biologic agents among rheumatoid arthritis patients enrolled in the US medicare program. Arthritis Care Res (Hoboken).

[23] Weinblatt ME, Schiff M, Valente R, van der Heijde D, Citera G, Zhao C, et al. Head-to-head comparison of subcutaneous abatacept versus adalimumab for Rheumatoid Arthritis. Arthritis Rheum. 2013;65(1):28–38. 10.1002/art.37711 PMC3572583 23169319

[24] Schiff M, Keiserman M, Codding C, Songcharoen S, Berman A, Nayiager S (2008). Efficacy and safety of abatacept or infliximab vs placebo in ATTEST: a phase III, multi-centre, randomised, double-blind, placebo-controlled study in patients with rheumatoid arthritis and an inadequate response to methotrexate. Ann Rheum Dis.

[25] Westhovens R, Yocum D, Han J, Berman A, Strusberg I, Geusens P (2006). The safety of infliximab, combined with background treatments, among patients with rheumatoid arthritis and various comorbidities: a large, randomized, placebo-controlled trial. Arthritis Rheum.

[26] Wailoo AJ, Bansback N, Brennan A, Michaud K, Nixon RM, Wolfe F (2008). Biologic drugs for rheumatoid arthritis in the Medicare program: a cost-effectiveness analysis. Arthritis Rheum.

[27] Yun H, Xie F, Beyl RN, Chen L, Lewis JD, Saag KG (2017). Risk of hypersensitivity to biologic agents among Medicare patients with rheumatoid arthritis. Arthritis Care Res (Hoboken)..

[28] Janssen Biotech, Inc v. Celltrion Healthcare Co., LTD. U.S. Court of Appeals for the Federal Circuit. https://www.bigmoleculewatch.com/wp-content/uploads/2016/12/Janssen-reply-for-extension.pdf. Accessed 11 Mar 2019.

[29] Rockoff JD. Pfizer Alleges J&J Thwarted Competition to Remicade, in Legal Test of Biotech-Drug Copies. In: The Wall Street Journal. 2017. https://www.wsj.com/articles/pfizer-files-antitrust-lawsuit-alleging-j-j-thwarted-use-of-biosimilar-rival-to-remicade-1505913080 Accessed 11 Mar 2019.

[30] U.S. Centers for Medicare & Medicaid Services (https://CMS.gov). Medicare Part B Drug Average Sales Price - Manufacturer reporting of Average Sales Price (ASP) data. 2019. https://www.cms.gov/Medicare/Medicare-Fee-for-Service-Part-B-Drugs/McrPartBDrugAvgSalesPrice/index.html. Accessed 11 Mar 2019.

