# Supplementary material for: Will savings from biosimilars offset increased costs related to dose escalation? A comparison of infliximab and golimumab for rheumatoid arthritis
Source: Arthritis Res Ther. 2019 Dec 12;21:285. doi: 10.1186/s13075-019-2022-8 (PMC6909454; doi:10.1186/s13075-019-2022-8)
Supplement: Supplementary file 1 — Additional file 1: Table S1. Baseline Patient and Physician Characteristics, stratified for infliximab vs. golimumab and by whether patients dose escalated*. SMD = standardized mean difference. A SMD > 0.10 (bolded) is indicative of a potentially important difference. Data shown as mean (standard deviation) or n (%). *Two consecutive infusions with a dose increase, or frequency increase, were required to satisfy this definition. **rather than hospital-based practice, research, or other/missing designations. Table S2. AHRQ CCS categories included in the inverse probability treatment weighting model to balance covariates between golimumab and infliximab initiators. [file 13075_2019_2022_MOESM1_ESM.docx]

**Appendix Table 1:** Baseline Patient and Physician Characteristics, stratified for infliximab vs. golimumab and by whether patients dose escalated*

|  | **Golimumab with dose escalation**  **N=139** | **Golimumab without dose escalation n=2704** | **SMD** | **Infliximab with dose escalation n=2,541** | **Infliximab without dose escalation n=2,633** | **SMD** |
| --- | --- | --- | --- | --- | --- | --- |
| **Physician-specific factors** |  |  |  |  |  |  |
| **Female sex** | 37 (26.6%) | 640 (23.7%) | 0.07 | 703 (27.7%) | 663 (25.2%) | 0.06 |
| **Age in years, Mean (STD)** | **54.23 (10.15)** | **52.65 (9.37)** | **0.16** | 50.98 (9.34) | 51.85 (9.24) | 0.09 |
| **Years in practice** | **22.54 (11.63)** | **20.64 (10.80)** | **0.17** | 18.22 (10.80) | 19.14 (10.83) | 0.09 |
| **Ownership of infusion center** | 121 (87.1%) | 2406 (89.0%) | 0.06 | 2090 (82.3%) | 2073 (78.7%) | 0.09 |
| **Office-based practice**** | **115 (82.7%)** | **2113 (78.1%)** | **0.12** | 1908 (75.1%) | 2002 (76.0%) | 0.02 |
| **Type of Employment** |  |  |  |  |  |  |
| **Federal government** | 4 (2.9%) | 104 (3.8%) | 0.05 | 113 (4.4%) | 78 (3.0%) | 0.08 |
| **Group practice** | 58 (41.7%) | 1206 (44.6%) | 0.06 | 1042 (41.0%) | 1134 (43.1%) | 0.04 |
| **Local government** | 8 (5.8%) | 177 (6.5%) | 0.03 | 221 (8.7%) | 210 (8.0%) | 0.03 |
| **Medical school** | 2 (1.4%) | 77 (2.8%) | 0.10 | 53 (2.1%) | 58 (2.2%) | 0.01 |
| **Other** | 33 (23.7%) | 563 (20.8%) | 0.07 | 617 (24.3%) | 599 (22.7%) | 0.04 |
| **Solo practice** | 34 (24.5%) | 577 (21.3%) | 0.07 | 495 (19.5%) | 554 (21.0%) | 0.04 |
| **Patient-specific factors** |  |  |  |  |  |  |
| **Demographics** |  |  |  |  |  |  |
| **Age in years, Mean (STD)** | 70.12 (8.60) | 70.01 (8.92) | 0.01 | 68.40 (9.19) | 68.90 (9.72) | 0.05 |
| **Female** | 117 (84.2%) | 2170 (80.3%) | 0.10 | 1929 (75.9%) | 2059 (78.2%) | 0.05 |
| **White** | 113 (81.3%) | 2236 (82.7%) | 0.04 | 2095 (82.4%) | 2138 (81.2%) | 0.03 |
| **Dual eligible for Medicare and Medicaid** | 17 (12.2%) | 331 (12.2%) | 0.00 | 403 (15.9%) | 483 (18.3%) | 0.07 |
| **Disability according to Medicare as original reason for Medicare eligibility** | 53 (38.1%) | 951 (35.2%) | 0.06 | 804 (31.6%) | 872 (33.1%) | 0.03 |
| **Comorbidity diagnoses, %** |  |  |  |  |  |  |
| **Myocardial infarction** | < 11 | 130 (4.8%) | 0.01 | 117 (4.6%) | 109 (4.1%) | 0.02 |
| **Coronary heart disease** | 24 (17.3%) | 530 (19.6%) | 0.06 | 471 (18.5%) | 506 (19.2%) | 0.02 |
| **Peripheral vascular disease** | **16 (11.5%)** | **201 (7.4%)** | **0.14** | 179 (7.0%) | 210 (8.0%) | 0.04 |
| **Chronic pulmonary disease** | **30 (21.6%)** | **741 (27.4%)** | **0.14** | 622 (24.5%) | 737 (28.0%) | 0.08 |
| **Peptic ulcer disease** | **< 11** | **47 (1.7%)** | **0.12** | 45 (1.8%) | 41 (1.6%) | 0.02 |
| **Diabetes** | 37 (26.6%) | 621 (23.0%) | 0.08 | 581 (22.9%) | 587 (22.3%) | 0.01 |
| **Renal disease** | **< 11** | **277 (10.2%)** | **0.11** | 219 (8.6%) | 243 (9.2%) | 0.02 |
| **Malignancy** | 15 (10.8%) | 180 (6.7%) | 0.15 | 163 (6.4%) | 168 (6.4%) | 0.00 |
| **Fibromyalgia** | 34 (24.5%) | 557 (20.6%) | 0.09 | 487 (19.2%) | 491 (18.6%) | 0.01 |
| **RA and other medications, %** |  |  |  |  |  |  |
| **Methotrexate** | 80 (57.6%) | 1562 (57.8%) | 0.00 | 1823 (71.7%) | 1772 (67.3%) | 0.10 |
| **Other conventional DMARDS** | **44 (31.7%)** | **1041 (38.5%)** | **0.14** | 1205 (47.4%) | 1181 (44.9%) | 0.05 |
| **Oral glucocorticoids** | 98 (70.5%) | 1941 (71.8%) | 0.03 | 1932 (76.0%) | 1982 (75.3%) | 0.02 |
| **NSAIDs** | 62 (44.6%) | 1237 (45.7%) | 0.02 | 1210 (47.6%) | 1243 (47.2%) | 0.01 |
| **Opioid** | 103 (74.1%) | 1927 (71.3%) | 0.06 | 1783 (70.2%) | 1802 (68.4%) | 0.04 |
| **Statin** | 63 (45.3%) | 1252 (46.3%) | 0.02 | 1088 (42.8%) | 1131 (43.0%) | 0.00 |
| **Other lipid lower drug** | 13 (9.4%) | 244 (9.0%) | 0.01 | 225 (8.9%) | 193 (7.3%) | 0.06 |
| **Anti-hypertensive drug** | 107 (77.0%) | 2095 (77.5%) | 0.01 | 1883 (74.1%) | 1939 (73.6%) | 0.01 |
| **Anti-depressant drug use** | 62 (44.6%) | 1221 (45.2%) | 0.01 | 1072 (42.2%) | 1109 (42.1%) | 0.00 |
| **Healthcare utilization** |  |  |  |  |  |  |
| **Number of physician visits, Mean (STD )** | 18.59 (9.12) | 17.86 (9.37) | 0.08 | 16.91 (9.02) | 17.00 (9.32) | 0.01 |
| **Any hospitalization** | 33 (23.7%) | 571 (21.1%) | 0.06 | 492 (19.4%) | 535 (20.3%) | 0.02 |
| **Colon cancer screening** | 22 (15.8%) | 420 (15.5%) | 0.01 | 476 (18.7%) | 455 (17.3%) | 0.04 |
| **Breast cancer screening** | **68 (48.9%)** | **1129 (41.8%)** | **0.14** | 1045 (41.1%) | 1052 (40.0%) | 0.02 |

SMD = standardized mean difference. A SMD > 0.10 (bolded) is indicative of a potentially important difference

Data shown as mean (standard deviation) or n (%)

*Two consecutive infusions with a dose increase, or frequency increase, were required to satisfy this definition

**rather than hospital-based practice, research, or other/missing designations

**Appendix Table 2:** AHRQ CCS categories included in the inverse probability treatment weighting model to balance covariates between golimumab and infliximab initiators

| CCS DIAGNOSIS CATEGORIES | CCS DIAGNOSIS CATEGORIES LABELS |
| --- | --- |
| 10 | Immunizations and screening for infectious disease |
| 114 | Peripheral and visceral atherosclerosis |
| 125 | Acute bronchitis |
| 127 | Chronic obstructive pulmonary disease and bronchiectasis |
| 13 | Cancer of stomach |
| 154 | Noninfectious gastroenteritis |
| 160 | Calculus of urinary tract |
| 164 | Hyperplasia of prostate |
| 166 | Other male genital disorders |
| 173 | Menopausal disorders |
| 181 | Other complications of pregnancy |
| 184 | Early or threatened labor |
| 185 | Prolonged pregnancy |
| 191 | Polyhydramnios and other problems of amniotic cavity |
| 193 | OB-related trauma to perineum and vulva |
| 198 | Other inflammatory condition of skin |
| 199 | Chronic ulcer of skin |
| 2 | Septicemia (except in labor) |
| 201 | Infective arthritis and osteomyelitis (except that caused by tuberculosis or sexually transmitted disease) |
| 202 | Rheumatoid arthritis and related disease |
| 206 | Osteoporosis |
| 207 | Pathological fracture |
| 208 | Acquired foot deformities |
| 209 | Other acquired deformities |
| 210 | Systemic lupus erythematosus and connective tissue disorders |
| 212 | Other bone disease and musculoskeletal deformities |
| 217 | Other congenital anomalies |
| 219 | Short gestation; low birth weight; and fetal growth retardation |
| 226 | Fracture of neck of femur (hip) |
| 229 | Fracture of upper limb |
| 231 | Other fractures |
| 235 | Open wounds of head; neck; and trunk |
| 239 | Superficial injury; contusion |
| 244 | Other injuries and conditions due to external causes |
| 245 | Syncope |
| 249 | Shock |
| 26 | Cancer of cervix |
| 2603 | E Codes: Fall |
| 2605 | E Codes: Firearm |
| 27 | Cancer of ovary |

**Appendix Table 2:** AHRQ CCS categories included in the logistic model for probability for golimumab vs. infliximab

| 29 | Cancer of prostate |
| --- | --- |
| 3 | Bacterial infection; unspecified site |
| 38 | Non-Hodgkin`s lymphoma |
| 40 | Multiple myeloma |
| 49 | Diabetes mellitus without complication |
| 50 | Diabetes mellitus with complications |
| 53 | Disorders of lipid metabolism |
| 55 | Fluid and electrolyte disorders |
| 58 | Other nutritional; endocrine; and metabolic disorders |
| 653 | Delirium, dementia, and amnestic and other cognitive disorders |
| 654 | Developmental disorders |
| 77 | Encephalitis (except that caused by tuberculosis or sexually transmitted disease) |
| 83 | Epilepsy; convulsions |
| 87 | Retinal detachments; defects; vascular occlusion; and retinopathy |
| 94 | Other ear and sense organ disorders |
| 98 | Essential hypertension |

**Supplemental Figure 1: Cohort Selection**

**Supplemental Figure 2: Title: Decrease in Average Selling Price* of Infliximab Biosimilars Over Time**

**Centers for Medicare and Medicaid CMS Medicare Part B Drug Average Sales Price Report (updated September 10, 2019 from https://www.cms.gov/Medicare/Medicare-Fee-for-Service-Part-B-drugs/McrPartBDrugAvgSalesPrice/2018ASPFiles.html)*
