# Supplementary figures and images for: Will savings from biosimilars offset increased costs related to dose escalation? A comparison of infliximab and golimumab for rheumatoid arthritis
Source: Arthritis Res Ther. 2019 Dec 12;21:285. doi: 10.1186/s13075-019-2022-8 (PMC6909454; doi:10.1186/s13075-019-2022-8)

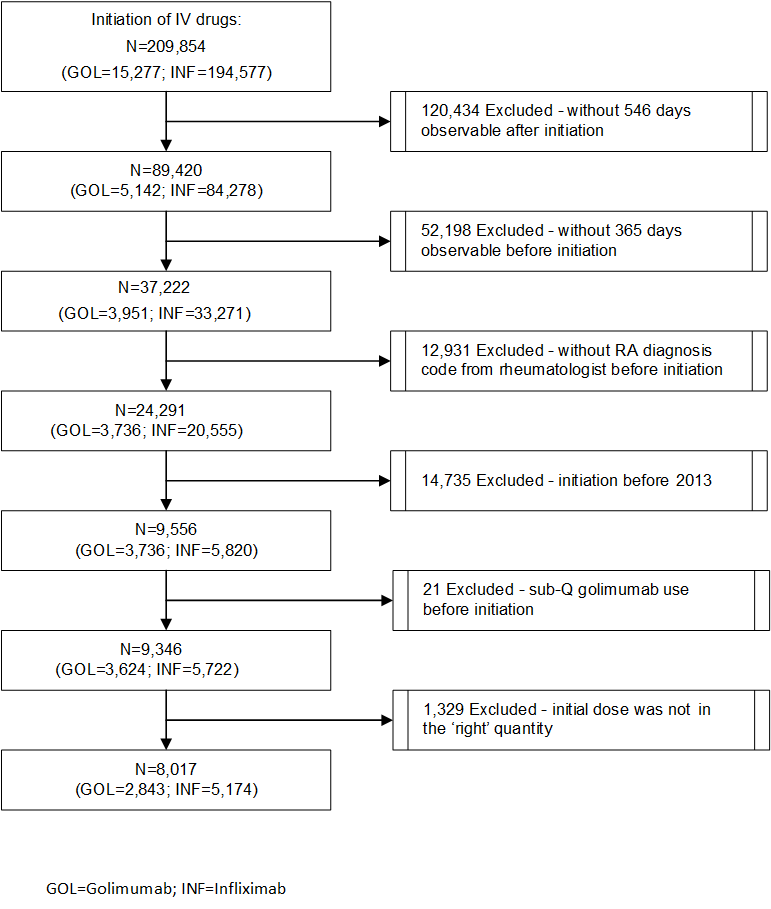

Supplement: Supplementary file 2 — Additional file 2: Figure S1. Cohort Selection. [file 13075_2019_2022_MOESM2_ESM.tif]

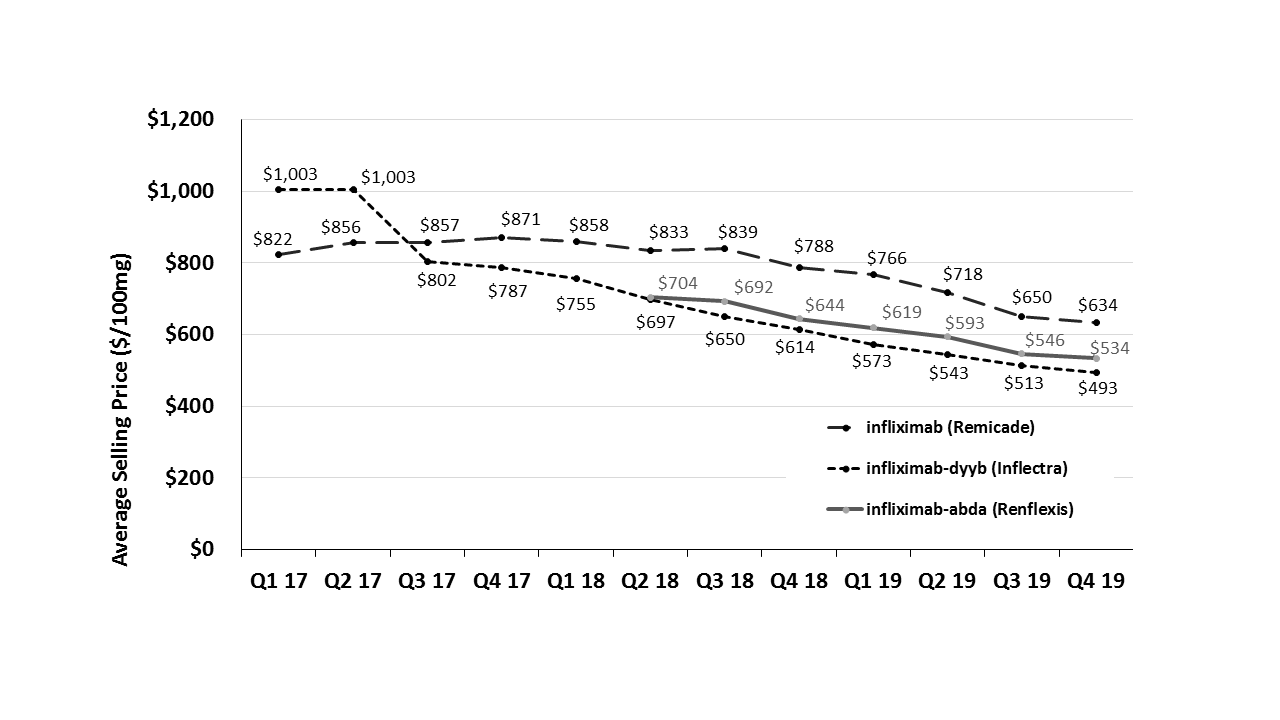

Supplement: Supplementary file 3 — Additional file 3: Figure S2. Decrease in Average Selling Price* of Infliximab Biosimilars Over Time. *Centers for Medicare and Medicaid CMS Medicare Part B Drug Average Sales Price Report (updated September 10, 2019 from https://www.cms.gov/Medicare/Medicare-Fee-for-Service-Part-B-drugs/McrPartBDrugAvgSalesPrice/2018ASPFiles.html). [file 13075_2019_2022_MOESM3_ESM.tif]
